# Phenotypic Screens Identify Genetic Factors Associated with Gametocyte Development in the Human Malaria Parasite Plasmodium falciparum

**DOI:** 10.1128/spectrum.04164-22

**Published:** 2023-05-08

**Authors:** Jyotsna Chawla, Ilana Goldowitz, Jenna Oberstaller, Min Zhang, Camilla Valente Pires, Francesca Navarro, Lauriane Sollelis, Chengqi C. Q. Wang, Andreas Seyfang, Jeffrey Dvorin, Thomas D. Otto, Julian C. Rayner, Matthias Marti, John H. Adams

**Affiliations:** a Department of Molecular Medicine, Morsani College of Medicine, University of South Florida, Tampa, Florida, USA; b Center for Global Health and Infectious Diseases Research, College of Public Health, University of South Florida, Tampa, Florida, USA; c Department of Immunology and Infectious Diseases, Harvard School of Public Health, Boston, Massachusetts, USA; d Boston Children’s Hospital and Harvard Medical School, Harvard Medical School, Boston, Massachusetts, USA; e Institute of Infection, Immunity, and Inflammation, College of Medical, Veterinary and Life Sciences, University of Glasgow, Glasgow, United Kingdom; f Institute of Parasitology Zurich, VetSuisse Faculty, University of Zurich, Zurich, Switzerland; g Cambridge Institute for Medical Research, University of Cambridge, Cambridge, United Kingdom; Hebrew University of Jerusalem

**Keywords:** functional genomics, *piggyBac*, insertional mutagenesis, gametocytogenesis, sexual development

## Abstract

Transmission of the deadly malaria parasite Plasmodium falciparum from humans to mosquitoes is achieved by specialized intraerythrocytic sexual forms called gametocytes. Though the crucial regulatory mechanisms leading to gametocyte commitment have recently come to light, networks of genes that control sexual development remain to be elucidated. Here, we report a pooled-mutant screen to identify genes associated with gametocyte development in P. falciparum. Our results categorized genes that modulate gametocyte progression as hypoproducers or hyperproducers of gametocytes, and the in-depth analysis of individual clones confirmed phenotypes in sexual commitment rates and putative functions in gametocyte development. We present a new set of genes that have not been implicated in gametocytogenesis before and demonstrate the potential of forward genetic screens in isolating genes impacting parasite sexual biology, an exciting step toward the discovery of new antimalarials for a globally significant pathogen.

**IMPORTANCE** Blocking human-to-vector transmission is an essential step toward malaria elimination. Gametocytes are solely responsible for achieving this transmission and represent an opportunity for therapeutic intervention. While these falciform-shaped parasite stages were first discovered in the 1880s, our understanding of the genetic determinants responsible for their formation and molecular mechanisms that drive their development is limited. In this work, we developed a scalable screening methodology with *piggyBac* mutants to identify genes that influence the development of gametocytes in the most lethal human malaria parasite, P. falciparum. By doing so, we lay the foundation for large-scale functional genomic studies specifically designed to address remaining questions about sexual commitment, maturation, and mosquito infection in P. falciparum. Such functional genetic screens will serve to expedite the identification of essential pathways and processes for the development of novel transmission-blocking agents.

## INTRODUCTION

Malaria is a major public health problem and a leading cause of morbidity and mortality caused by unicellular parasites of the genus *Plasmodium*. In 2020, an estimated 241 million people were infected with malaria and over half a million succumbed to this mosquito-borne infectious disease (1). About 95% of these deaths occurred in sub-Saharan Africa, where Plasmodium falciparum, the most virulent human malaria parasite species predominates (2). The emergence of parasite resistance to the frontline drug artemisinin and its spread to regions where malaria is endemic with high transmission rates are alarming (3, 4). To maintain the current efforts in malaria control, and to bolster the push for elimination in specific regions, there is a clear need for new drug therapies and efficacious multistage vaccines informed by a better understanding of the parasite’s biology.

P. falciparum has a complex life cycle spanning stages in both human and mosquito hosts. While the human disease is a manifestation of asexual parasite multiplication in red blood cells, sexual differentiation into gametocytes is crucial for transmission to the *Anopheles* mosquito vector. During each intraerythrocytic replication cycle, a subpopulation of parasites commits to sexual development (5), a switch that was found to be regulated by a conserved transcription factor AP2-G (6). *Ap2-g* activation results in expression of early gametocyte-specific genes that are indicators of commitment to sexual development (7, 8). Transmission-competent P. falciparum gametocytes are morphologically distinct from their asexual counterparts. They have undergone extensive development over a period of 10 to 12 days through a series of morphologically distinguishable stages, designated I to V (9, 10). A large portion of gametocyte proteins are thus exported early during development (25 to 32 h postinfection) to catalyze the extensive host cell remodeling (11). In addition, metabolic processes such as mitochondrial respiration and production of membrane lipids are accelerated to meet the requirements of the developing gametocytes (12, 13). For the most part, immature gametocyte stages I to IV are retained in the extravascular niche of the bone marrow due to their mounting rigidity, while the deformable stage V gametocytes intravasate into circulation, where they are picked up by the mosquito during a blood meal (14–16). Successful fusion of a male and female gamete drives onward sexual development, establishing an infection in the mosquito vector (17). The genetic factors underlying sexual development, sex ratio determination, bone marrow sequestration, cytoskeletal dynamism, and other cues for metabolic redirection remain largely unknown and offer critical avenues for transmission-blocking drug discovery and vaccine development.

Recent transcriptomic, proteomic, and metabolomic approaches have identified important sexual-stage signatures (7, 18–20). However, many of the identified genes have no homologs in other well studied organisms, making it difficult to interpret biological function based on sequence alone (21). Most attempts to understand gene function during gametocytogenesis have employed targeted reverse-genetic approaches that genetically disrupt a gene of interest to analyze its phenotype (22). These efforts have revealed genes essential for development in the mosquito stages (23–28). They have also only assessed a relatively small number of the hundreds of genes that are suggested by omics data to have functions during gametocytogenesis, but most remain to be characterized. Our understanding of molecular mechanisms governing sexual development in P. falciparum has been mainly constrained by the technical difficulties with culturing gametocytes, lack of detectable homology, and the hurdles associated with gene-by-gene approaches, where multiple attempts are often required before a successful mutant is obtained.

Insertional mutagenesis using the *piggyBac* (*pB*) transposon is a robust alternative to overcome limitations in functional annotation of P. falciparum genes (29). The *piggyBac* randomly inserts at TTAA sites, which are abundantly present in the parasite’s AT-rich genome (about every 70 to 80 bp), and depending on where the insertion occurs, it can either disrupt or modulate gene function (30). Previously, we used the *piggyBac* system to generate a saturation library of 38,000 single-disruption mutants while also classifying genes as either essential or dispensable for blood-stage survival of the parasite (31). Of the 2,042 genes determined to be dispensable during blood-stage growth (40% of the genome), many of them showed peak expression in gametocytes (~800 genes), including the master regulators of sexual commitment and leading transmission-blocking vaccine (TBV) candidates (31, 32). This apparent abundance of critical sexual-stage genes among genes dispensable for asexual development prompted us to investigate genetic factors involved in gametocyte development with a forward genetic approach.

Pooled genetic screens offer the advantages of rapid, high-throughput, and unbiased screening of genomic elements influencing selected phenotypes and have been successfully applied in other apicomplexans (33–37). Recently, we developed a large-scale screening methodology using *piggyBac* mutants to decode the parasite processes essential for survival in malarial febrile temperatures (38, 39). Here, we report a scalable phenotypic screening strategy with isogenic *piggyBac* mutants for identification of genetic factors associated with gametocytogenesis in P. falciparum. We assessed a population of mutants (*n* = 128) for their ability to complete gametocyte development and categorized them as hypoproducers or hyperproducers of gametocytes based on relative depletion/enrichment. We performed a secondary gametocyte conversion screen with individual *piggyBac* mutant clones (*n* = 60) and established a catalog of shared hits with consistent phenotypes across the screens. We also report the involvement of two new genes in gametocyte development, by means of additional phenotypic characterization. This study provides the proof of concept necessary for future large-scale genetic screens aimed at uncovering the fundamentals of *Plasmodium* sexual development.

## RESULTS

### A pooled screen of *a piggyBac* mutant library allows identification of gametocyte phenotypes.

We used a library of 128 well-characterized single-disruption *piggyBac* mutants with insertions spanning coding sequences, untranslated regions, and intergenic regions, representing genes coding for proteins, noncoding RNA (ncRNA), and pseudogenes (see Fig. S1 in the supplemental material). Earlier, we demonstrated that the growth phenotypes of individual mutant parasite lines can be accurately distinguished in pooled screening by quantitative insertion site sequencing (QI-seq), a high-throughput sequencing method that identifies a *piggyBac* insertion site and quantifies each mutant in the library at any given time (40, 41). Thus, increase or decrease in abundance of individual mutants can be tracked to associate an individual genetic mutant with the phenotype in question. We hypothesized that genes essential for the gametocytes would be identifiable through decreased relative abundance after gametocyte development.

**FIG 1:**
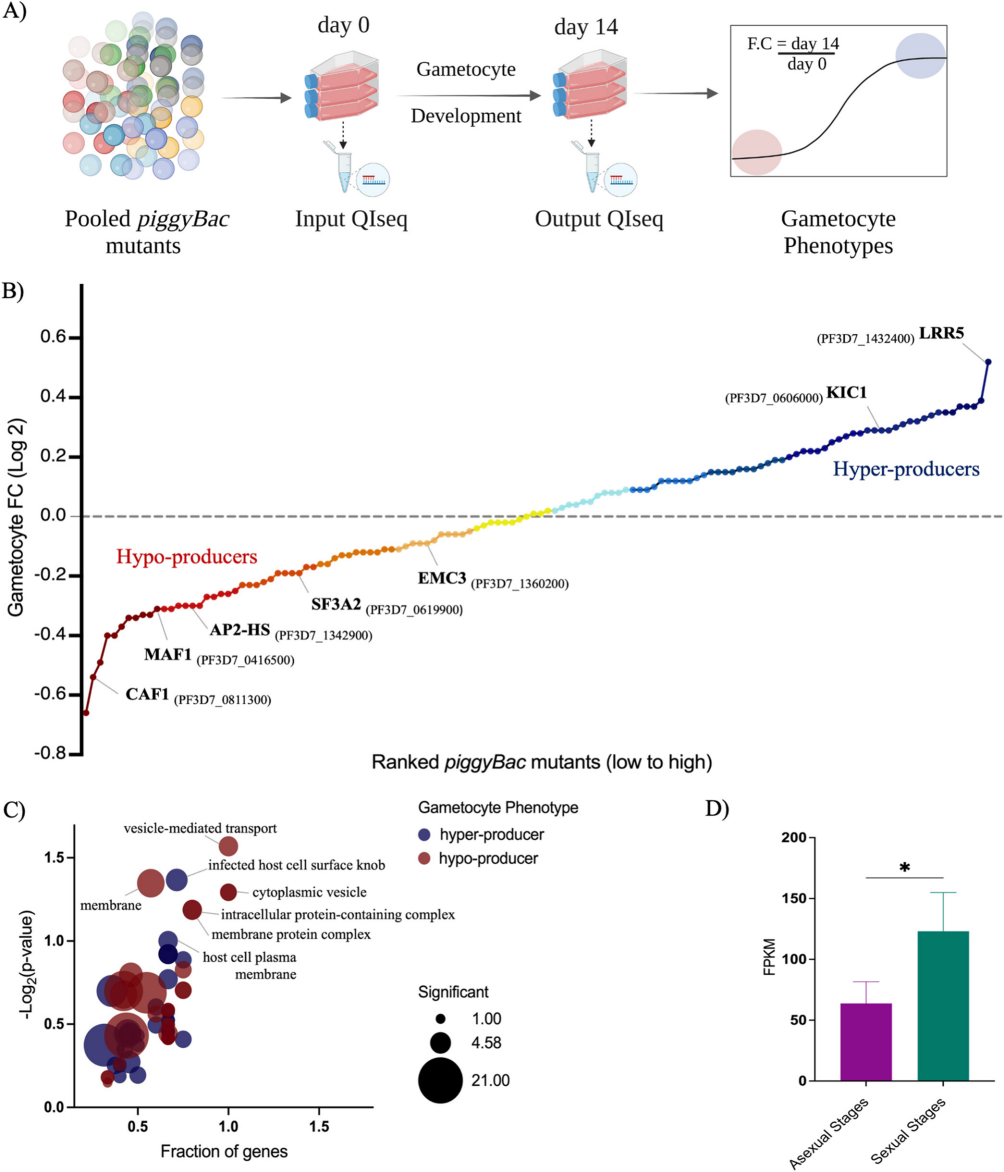
A pooled screen of P. falciparum *piggyBac* mutants allows identification of gametocyte phenotypes. (A) Experimental design for the pooled gametocyte phenotypic screen. An extensively characterized pool of *piggyBac* mutant clones (*n* = 128) was induced for gametocyte development *in vitro* by routine culturing (see Materials and Methods). Parasite genomic DNA was harvested at day 0 and day 14 (>90% mature gametocytes). QI-seq quantified each *piggyBac* mutant in the library by sequencing from the 5′ ends of a *piggyBac* insertion site (40). High correlation of normalized reads for the two time points was observed across the three biological replicates, indicating high accuracy and reproducibility of sequencing data (Fig. S2). (The schematics were created using BioRender.com.) (B) Ranked *piggyBac* mutants based on gametocyte phenotypes in the library. QI-seq results rely on counts of insertion sites for each mutant, which are normalized to calculate the relative abundances at day 14 and day 0. Gametocyte fold change (day 14/day 0) was calculated for each mutant as a measure of their ability to differentiate into mature gametocytes. The *piggyBac* mutants ranked at the bottom of the curve (log_2_ FC < −0.1) were classified as hypoproducers (*n* = 46) of gametocytes, and those ranked at the top (log_2_ FC > 0.1) were classified as hyperproducers (*n* = 48) of gametocytes. (C) Functional enrichment of significant GO terms for gametocyte hypoproducer and hyperproducer *piggyBac* mutants versus all other mutants in the library (*n* = 128). Circles represent the GO terms, circle color represents the assigned gametocyte phenotypic category (red for hypoproducers and blue for hyperproducers), and circle size represents the number of significant genes annotated to that category (see Materials and Methods). Gametocyte hypoproducers were enriched in terms associated with membrane, cytoplasmic vesicle, and vesicle-mediated transport, while hyperproducers tended to be enriched for GO terms like host cell plasma membrane and infected host cell surface knob (two-tailed Fisher/elim-hybrid test *P* value ≤ 0.1). (D) The mean number of fragments per kilobase per million (FPKM) of all the hypo- and hyperproducer genes was determined from published transcriptome sequencing (RNA-seq) data (Data Set S2) (mean and standard errors of the means [SEM]) (48). The expression of these phenotyped genes during sexual stages (II, V, and ookinete) were higher than in asexual stages (ring, trophozoite, and schizont), suggesting a potential role in sexual development. *, *P* < 0.05 (Mann-Whitney U test).

The *piggyBac* mutant pool (also called the pilot library) was subjected to *in vitro* culture conditions favoring gametocytogenesis and allowed to develop for 14 days until a bulk of mature stage V gametocytes (>90%) was observed. Genomic DNA was harvested at day 0 (before induction of gametocytogenesis) and day 14 (mature gametocytes and endpoint of the experiment), and the proportions of mutants at both time points were quantified by QI-seq (Fig. 1A). Mutants with a lower abundance on day 14 had fewer QI-seq reads than at their starting point at day 0 and were categorized as hypoproducers of gametocytes (46 genes with a log fold change [FC] less than −0.1). The mutants with higher abundance on day 14 had more QI-seq reads and were classified as hyperproducers of gametocytes (48 genes with log fold change of >0.1), while the unclassified mutants between these groups were considered neutral (*n* = 33) (Fig. 1B; Data Set S1).

Some of the hits identified as hypoproducers in our results (highlighted in Fig. 1B) were previously linked to deleterious gametocyte phenotypes in independent studies. These include mRNA regulators like CAF1, translational repressors like Maf1 and a transcription factor AP2-HS of the highly conserved ApiAP2 family (42–44). The hypoproducer phenotypic group was enriched for gene ontology (GO) terms like RNA and protein binding, membrane, intracellular protein complex, and, interestingly, vesicle-mediated transport (Fig. 1C; Data Set S2). While the importance of protein-RNA interactions in gametocyte regulation has been known for some time, the critical nature of the gametocyte inner membrane complex emerged only recently (45–47). The *piggyBac* mutants assigned to either of the gametocyte phenotype categories generally represented disruptions in genes that had increased expression in sexual stages compared to asexual blood stages, indicating a potential role of these genes in parasite sexual development (Fig. 1D; Data Set S2) (48). Furthermore, we characterized 17 conserved *Plasmodium* proteins with previously unknown functions as having a gametocyte phenotype (10 hypoproducer and 7 hyperproducer), aiding the process of functional annotation of these unexplored genes (Data Set S3).

### Initial characterization of individual *piggyBac* mutants determines gametocyte conversion rates.

Gametocyte commitment is the first step toward sexual development in P. falciparum (5, 10). Gametocyte conversion rate (GCR) is defined as the ratio of sexually committed parasites to the total number of parasites during a given intraerythrocytic cycle. We reasoned that hypoproducers in the pooled screen could have (i) reduced gametocyte conversion rates or (ii) a defect that stalls gametocyte development before stage V; meanwhile, hyperproducers in the pooled screen could have increased gametocyte conversion rates.

**FIG 2:**
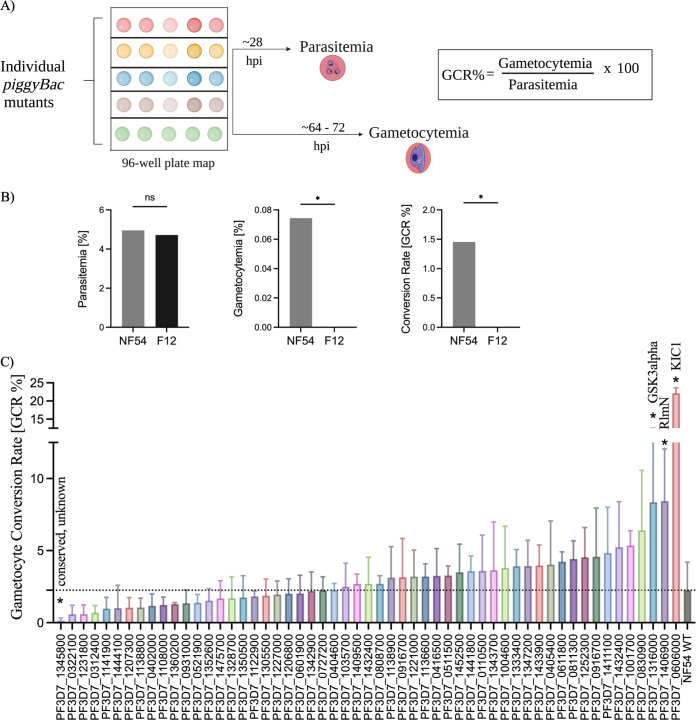
A cloned screen of P. falciparum *piggyBac* mutants determines gametocyte conversion rates. (A) Experimental design for the cloned gametocyte phenotypic screen. Individual *piggyBac* mutant clones and their parent strain, wild-type NF54, were thawed and subjected to a well-established sexual conversion assay with slight modifications (see Materials and Methods). Briefly, double sorbitol-synchronized parasite cultures were seeded at 1% parasitemia in 96-well plates. After about 24 to 28 h postinvasion, percent parasitemia for each well was determined using a DNA-labeling dye (SYBR green) on a flow cytometer. The percent gametocytemia from each well was measured 64 to 68 h postinvasion by immunofluorescence staining with an early-stage-gametocyte-specific marker Pfg27 (see Materials and Methods). All experiments were performed in five replicates. (The schematics were created using BioRender.com.) (B) The parameters of the assay were first tested with wild-type NF54 and the gametocyteless derivative line F12 as a negative control. The GCR was calculated as [(% gametocytemia/% parasitemia) × 100]. (C) GCRs from five replicates are plotted for the individual *piggyBac* mutant clones (*n* = 55) and wild-type NF54 (data are means and SD). Four *piggyBac* mutants were significantly different from the full library. Three of the hits, PF3D7_0606000 (KIC1), PF3D7_140690 (radical SAM protein, putative), and PF3D7_1316000 (GSK3α, putative) had an increased gametocyte conversion rate, and one *piggyBac* mutant, PF3D7_1345800 (conserved *Plasmodium* protein, unknown function), had a decreased gametocyte conversion rate. *, *P* < 0.05 (one-way ANOVA and nonparametric Mann-Whitney test); ns, not significant. The dotted line represents the mean GCR of wild-type NF54.

To tease out these possibilities and introduce another layer of characterization through an early gametocyte time point, we subjected random individual *piggyBac* clones to a flow cytometry-based sexual conversion assay. Briefly, tightly synchronized *piggyBac* mutant clones were cultured individually, seeded at 1% parasitemia, and induced to form gametocytes (Fig. S4A), followed by determination of asexual parasitemia (20 to 24 h hours post-invasion [hpi]) and gametocytemia (64 to 72 hpi) to enable calculation of gametocyte conversion rates [% GCR = (% gametocytemia/% parasitemia) × 100] (Fig. 2A). The parameters of the assay were first optimized with the *piggyBac*-isogenic wild-type parental line NF54 and a gametocyte-deficient line, F12, as the negative control (Fig. 2B) (49).

In the cloned library screen, the mean gametocyte conversion rate of NF54 was around 2.25%, with all the other *piggyBac* mutant clones ranked from low to high (Fig. 2C). Three *piggyBac* mutants with insertions in the genes PF3D7_0606000 (KIC1), PF3D7_1406900 (radical *S*-adenosylmethionine [SAM] protein, putative), and PF3D7_1316000 (glycogen synthase kinase-3α [GSK3α], putative) had significantly increased gametocyte conversion rates (3- to 9-fold), while the *pB* mutant corresponding to the gene PF3D7_1345800 (conserved *Plasmodium* protein, unknown function) had a significantly (0.06-fold) decreased GCR, (Fig. 2C; Data Set S4). We observed consistent phenotypes across screens for 15 clones. The shared hits included 7 clones that had a GCR lower than the wild-type GCR and were gametocyte hypoproducers and 8 clones that had a GCR higher than the wild-type GCR and were gametocyte hyperproducers (Table 1). Some *pB* mutants (*n* = 15) had a gametocyte conversion rate higher than that of the wild type but were hypoproducers in the pooled screen. This suggested that the disrupted genes are essential for the development of later gametocyte stages, as detected in the pooled screen, but not for commitment or early development. In support of this idea, the published RNA sequencing data show that these genes had significantly higher levels of expression in stage V gametocytes than in stage II gametocytes (Fig. S4B). We found a weak correlation between parasitemia and gametocytemia of the *piggyBac* clones under study (Fig. S4C).

**TABLE 1:** Top gametocyte hits from *piggyBac* phenotypic screens

Gene ID	Description	Gametocyte phenotype	
		Stage V^*a*^	Conversion^*b*^
PF3D7_1360200	EMC3, putative	Hypo	GCR*_pB_* < GCR_WT_
PF3D7_1207300	Lysosomal integral membrane protein (LIMP), putative	Hypo	GCR*_pB_* < GCR_WT_
PF3D7_0402000	*Plasmodium* exported protein (PHISTa), unknown function	Hypo	GCR*_pB_* < GCR_WT_
PF3D7_1227000	Conserved *Plasmodium* protein, unknown function	Hypo	GCR*_pB_* < GCR_WT_
PF3D7_1206800	Conserved *Plasmodium* protein, unknown function	Hypo	GCR*_pB_* < GCR_WT_
PF3D7_1328700	*Plasmodium* RNA of unknown function (RUF1)	Hypo	GCR*_pB_* < GCR_WT_
PF3D7_1342900	AP2 domain transcription factor (AP2-HS)	Hypo	GCR*_pB_* < GCR_WT_
PF3D7_0404600	Conserved *Plasmodium* membrane protein, unknown function	Hyper	GCR*_pB_* > GCR_WT_
PF3D7_0511500	RNA pseudouridylate synthase, putative	Hyper	GCR*_pB_* > GCR_WT_
PF3D7_1432400	LRR5	Hyper	GCR*_pB_* > GCR_WT_
PF3D7_1138900	Noncoding RNA	Hyper	GCR*_pB_* > GCR_WT_
PF3D7_1347200	Nucleoside transporter 1 (NT1)	Hyper	GCR*_pB_* > GCR_WT_
PF3D7_1004600	Conserved *Plasmodium* membrane protein, unknown function	Hyper	GCR*_pB_* > GCR_WT_
PF3D7_1316000	GSK3α, putative	Hyper	GCR*_pB_* > GCR_WT_****
PF3D7_0606000	KIC1	Hyper	GCR*_pB_* > GCR_WT_****

a Phenotype determined in the pooled screen. Hypo, gametocyte hypoproducer; Hyper, gametocyte hyperproducer.

b GCR, gametocyte conversion rate; WT, wild-type NF54. “GCR*_pB_* < GCR_WT_” indicates that the mean GCR of the *piggyBac* mutant was lower than that of wild-type NF54, and “GCR*_pB_* > GCR_WT_” indicates that the mean GCR of the mutant was higher than that of the wild type. ****, significant GCR hit.

### Investigation of the ability of mutant parasites to complete gametocyte maturation.

**FIG 3:**
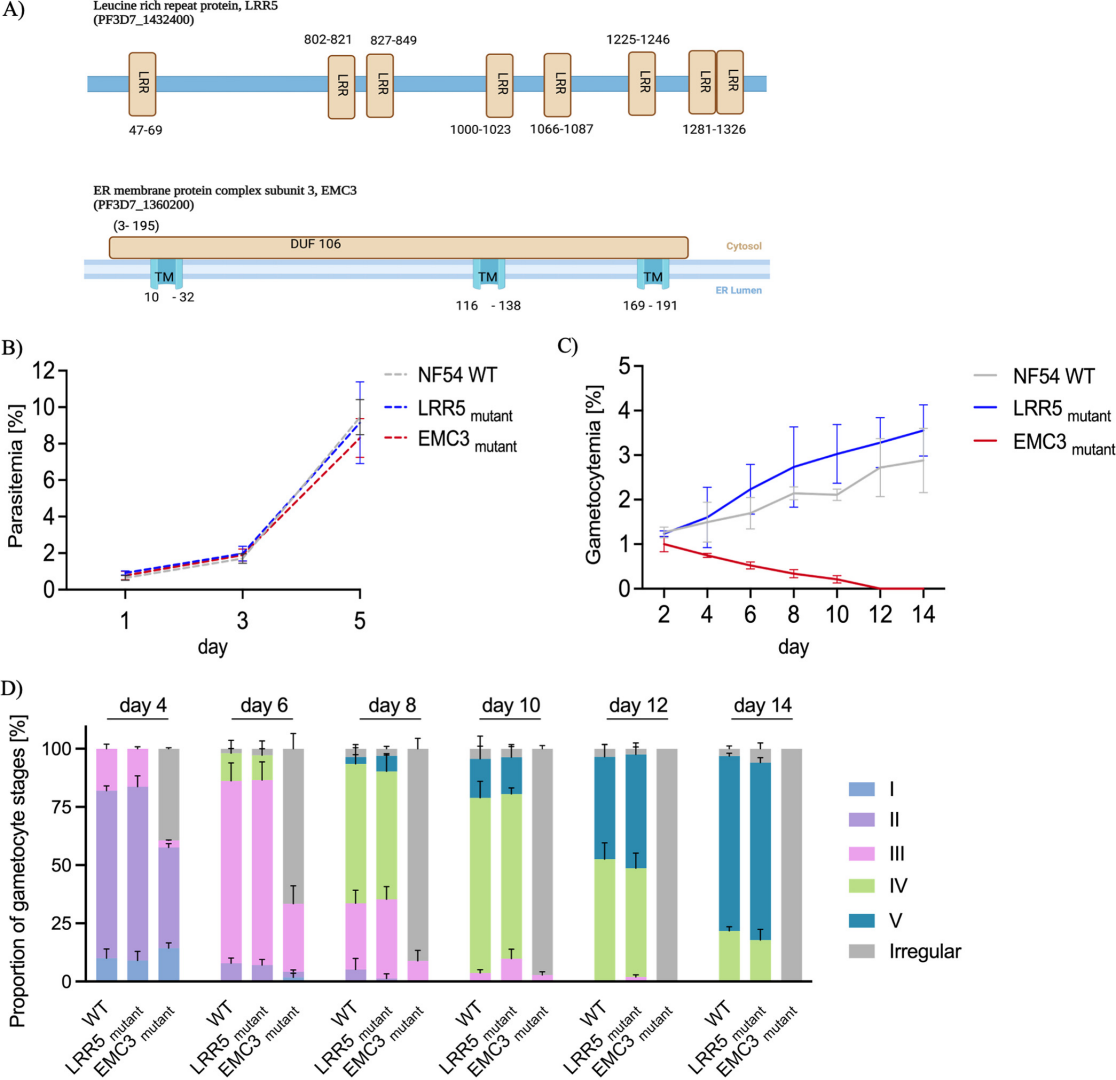
Additional phenotypic characterization of two high-confidence gametocyte hits. (A) Two gene candidates (EMC3 and LRR5) from the hypo- and hyperproducer gametocyte phenotypic categories, respectively, were subjected to further characterization and validation. The schematics of EMC3 and LRR5 show conserved functional domains The domains and motifs represented in the cartoon are not to scale. EMC3 has three transmembrane regions and one domain of unknown function with a putative ER localization signal. LRR5 has multiple leucine repeat domains (based on prediction analysis from ExPASy Prosite; graphic created using BioRender.com). (B) The asexual multiplication rates of the two *pB* mutants, the EMC3 and LRR5 mutants, were not different from that of wild-type NF54 (*P* > 0.05). Highly synchronized cultures of parasite lines were set at an initial parasitemia of 0.25% and monitored by using Giemsa smears for 2 growth cycles. Percent parasitemia was determined for three biological replicates by counting 2,000 RBCs each (means and SD [error bars] are shown). (C) Gametocyte development of the *pB* mutants and NF54 over a period of 14 days for three biological replicates (means with SD [error bars] are shown). The hyperproducer LRR5 mutant had increased numbers of gametocytes for all time points compared to the wild type (paired *t* test, *P* < 0.05). Meanwhile, the hypoproducer EMC3 mutant showed a rapid decline in gametocyte numbers compared to NF54 (paired *t* test, *P* < 0.05). (D) Proportions of gametocyte stages from days 4 to 14 for three biological replicates, as assessed by morphological classification from Giemsa-stained smears. Aberrant or deformed gametocytes that could not be categorized in one of the five developmental stages (I to V) were classified as “irregular” (gray bars). The development of gametocytes in LRR5 mutant was like that in the wild type, while the EMC3 mutant had increased numbers of malformed gametocytes from day 4 (two-way ANOVA, *P* < 0.05). No viable mature gametocytes were detected for the EMC3 mutant. (E) IFA of the EMC3 mutant confirmed impaired gametocyte development and revealed a developmental arrest at stage III. Antibodies against PfGAP45, a marker of the IMC that extends along the periphery during gametocyte morphogenesis (488 channel), were used to study morphology. (Top) Expected gametocyte developmental stages as observed in wild-type NF54; (bottom) impaired development of the IMC leading to irregular shapes in the *piggyBac* EMC3 mutant. Bars, 3 μm.

To validate the phenotype-genotype associations observed in the screens, we decided to pursue two *piggyBac* mutants on their path to gametocyte development. The gene candidates were short-listed from the list of top hits (Table 1), and selection was guided by additional criteria like evolutionary conservation and expression in gametocytes (Fig. S5; Data Set S6). This included a hypoproducer, PF3D7_1360200 (endoplasmic reticulum [ER] membrane protein complex subunit 3 [EMC3]), with a *piggyBac* insertion in its 3′ untranslated region (UTR) and a hyperproducer, PF3D7_1432400 (leucine rich repeat protein [LRR5]), with an exonic *piggyBac* insertion (Fig. 1B; Data Set S1). Bioinformatic domain prediction based on protein sequence showed three transmembrane regions and a conserved domain of unknown function for EMC3 (257 amino acids [aa]), while the relatively large protein LRR5 (1,504 aa) exhibited multiple leucine-rich repeat regions (~8) in its domain architecture (Fig. 3A) (50, 51).

We first evaluated the asexual growth of the *piggyBac* mutants. Briefly, highly synchronized ring stage cultures of the mutants and wild-type NF54 parasite line were observed for 5 days by using Giemsa-stained blood smears. There was no significant difference in the intraerythrocytic replication cycles between the two *pB* mutants and NF54 (Fig. 3B; Data Set S5). To validate gametocyte phenotypes and discern the nature of the hypoproducer phenotype in the EMC3 mutant, we monitored the gametocyte numbers and morphology for 14 days. The results showed an increased number of gametocytes throughout development for the LRR5 mutant (~1.2 times that of wild-type NF54), which agreed with its categorization as a gametocyte hyperproducer (Fig. 3C; Data Set S5). The hypoproducer EMC3 mutant showed a rapid decline in gametocyte numbers from day 4 and failed to complete gametocyte maturation (Fig. 3C; Data Set S5).

Further examination of gametocyte stage (I to V) distribution revealed a developmental arrest at stage III for the EMC3 mutant, beyond which atypical/pyknotic forms of gametocytes were observed (Fig. 3D; Data Set S5). The observed impaired gametocyte development was confirmed by superresolution immunofluorescence confocal microscopy (Fig. 3E). The inner membrane complex (IMC) is crucial for the metamorphosis of gametocytes (52). By immunofluorescence, we saw distorted and discontinuous IMC staining for PfGAP45, a key protein of the glideosome complex essential for gametocyte membrane assembly (52, 53). Improper IMC formation and subsequential aberration in gametocyte development has also been reported elsewhere (47). In all, these data highlight a role of EMC3 in gametocyte development and support the gametocyte phenotype outcomes assigned in the screens.

## DISCUSSION

Gametocytes produced in the human host after an active infection can persist and continue to serve as an infectious reservoir contributing to the spread of malaria. The development of new antimalarials and robust vaccines targeting these highly specialized precursor sex cells is imperative. Transposon-mediated insertional mutagenesis is a powerful technique to elucidate gene functions. The combined *pB*–QI-seq methodology enabled genome-level mutagenesis in the P. falciparum NF54 parasite line, defining genes as either essential or dispensable for asexual intraerythrocytic growth under ideal culture conditions (31). Previously, we demonstrated success in large-scale phenotypic screening for identification of genetic factors essential for parasite survival under heat shock stress (39). In this study, we established the methodology to identify genetic factors influencing gametocyte development using the robust and accurate *pB*–QI-seq approach.

Based on the rationale that a gene’s functional importance is evident under certain conditions, we subjected pools of *pB* mutants to gametocytogenesis *in vitro* and classified the depleted population after gametocyte selection as hypoproducers and the enriched population as hyperproducers. Of note, hits from the hypoproducer category included genes that have been reported to be deleterious to gametocytes (highlighted in Fig. 1B). One such example is CCR4-associated factor 1 (CAF1; PF3D7_0811300) of the CAF1/CCR4/NOT RNA metabolic complex, where disruption of CAF1 function in P. falciparum led to decreased gametocytemia as well as male gamete exflagellation (42). Specifically, the deadenylase domain of CAF1 was shown to be critical for its function in regulating gametocyte specific mRNA abundance (42).

In a previous forward genetic study that used *piggyBac* insertional mutagenesis for identification of parasite sexual-stage genes, Ikadai et al. identified several gametocyte-deficient mutants (43). One of their hits, a repressor of RNA polymerase III (Maf1; PF3D7_0416500), was shown to be essential for sexual differentiation. Not surprisingly, our *pB* mutant of the Maf1 gene carrying an insertion in the 5′ UTR was categorized as a hypoproducer of gametocytes. The authors also highlighted the role of splicing factor 3A subunit 3 (SF3A3), an important component of the 17S U2 small nuclear ribonucleoprotein alternative splicing complex, in early gametocyte development (43). The *piggyBac* mutant with a transposon insertion in another subunit of the same complex, known as splicing factor 3A subunit 2 (SF3A2) (putative; PF3D7_0619900), exhibited the hypoproducer phenotype in our screen, accentuating the role of mRNA splicing in sexual differentiation of P. falciparum gametocytes (Fig. 1B) (54).

More recently, a CRISPR-Cas9-mediated functional screen performed in the NF54 strain annotated several AP2 transcription factors with gametocyte phenotypes. Reduced gametocyte production and decreased abundance of the master regulator of sexual commitment (*pfap2-g*) in the AP2-HS (PF3D7_1342900) mutant were observed (44). Consistent with this finding, the *piggyBac* mutant for the AP2-HS transcription factor emerged as a gametocyte hypoproducer in our screens (Fig. 1B). The overlap in sexual-stage phenotypes described in other works and those assigned in this study indicates a strong corroboration between targeted functional studies and the high-throughput screening strategies.

Enhanced gametocyte conversion has been observed in response to different environmental stimuli (55–59). The phenotypic category of hyperproducers assigned in the pooled screen provides an opportunity to explore genetic determinants of increased gametocytogenesis. The *piggyBac* mutant for PF3D7_0606000 (K1C1) with a disruption in the 5′ UTR had a significantly increased gametocyte conversion rate (mean GCR, ~22%) in the clonal screen and was designated a hyperproducer in the pooled screen. K1C1 was recently identified as an interacting partner of the parasite’s K13 Kelch propeller protein, and although its role in artemisinin resistance is yet to be determined (60), it may be involved in stress response pathways that also regulate gametocytogenesis. New evidence on the transmission of parasites resistant to artemisinin and the impact of its derivative, dihydroartemisinin (DHA), on sexual conversion raises the possibility of a link between increased gametocytogenesis and drug resistance (61–64). The observation of increased gametocyte conversion for PF3D7_1406900, a putative radical SAM (RlmN), which is a key enzyme for transmethylation reactions, aligns with current research (65). A recent study highlighted the role of SAM in regulation of sexual differentiation by maintaining the histone methylation responsible for silencing *pfap2-g* in asexual parasite stages (66). The *piggyBac* disruption of SAM in our mutant could result in derepression of the sexual master regulator and a subsequent hyperproducer gametocyte phenotype. While the exact role of another hyperproducer, PF3D7_1316000 (PfGSK3α), needs to be investigated, its isoform, PfGSK3β, was recently shown to be essential for gametocyte development and maturation (67).

Additional characterization of two new genes, EMC3 (PF3D7_1360200) and LRR5 (PF3D7_1432400), identified via pooled screening as hypo- and hyperproducers, respectively, substantiates their role in gametocyte development. The highly conserved ER membrane complex (EMC) functions as an insertase in the ER membrane and facilitates membrane protein folding and assembly (68, 69). Extensive membrane development is one of the hallmarks of gametocytogenesis in P. falciparum, and the EMC has been shown to be involved in phospholipid synthesis in malaria parasites (46, 52, 70, 71). The inability to complete gametocyte maturation in the *piggyBac* mutant EMC3 and the absence of well-defined membrane structures emphasize a role of this gene in sexual development; nevertheless, additional studies are needed to validate gene function. The disruption in the 3′ UTR of the *piggyBac* mutant EMC3 may impact regulatory mechanisms crucial for sexual development, as seen previously for other sexual-stage genes (72–78).

Meanwhile, the gametocyte hyperproducer LRR5 (PF3D7_1432400), also a conserved gene, was shown to have relative peak expression in the late trophozoite stage, the stage at which parasites commit to sexual development (79, 80). In P. falciparum, leucine-rich-repeat proteins have been shown to facilitate protein-protein interactions, essential for regulation of the cell cycle through modulation of phosphatase activity (81). It is tempting to speculate that LRR5 might be involved in signal transduction pathways critical for gametocytogenesis. It is unknown whether enhanced gametocytogenesis also leads to increased mosquito infections, which was beyond the scope of this study.

Interestingly, proteins of genes disrupted in both hypo- and hyperproducer phenotypic categories had higher levels of conservation than those disrupted in parasites with neutral phenotypes (Fig. S3A). This high conservation of gametocyte-critical genes across *Plasmodium* species may allow extension of our analyses to other important malaria parasites, such as Plasmodium vivax, which lacks an *in vitro* culture system, and may further allow insight into the transmission biology of emerging reverse-zoonotic human malaria pathogens, such as Plasmodium knowlesi. While most gametocyte-critical genes were conserved, we observed 9 genes that had no orthologs and were specific to P. falciparum (Fig. S3B; Data Set S3), emphasizing the unique nature of P. falciparum gametocytogenesis (46).

Deeper knowledge of parasite sexual biology and the genes driving the development of transmission-competent gametocytes would have a significant advantage in the development of novel transmission-blocking agents. In this study, we established and validated a forward genetic screening approach for identifying essential sexual-stage genes that can be taken to the genome scale. Additional time points and environmental conditions can be introduced to explore the complex network of gametocyte-essential genes and pursue outstanding fundamental questions about sexual development in the malaria parasite P. falciparum.

## MATERIALS AND METHODS

### Parasite culture and maintenance.

The *pB* mutant lines and wild-type (WT) NF54 were cultured at 5% hematocrit (O^+^ erythrocytes from Interstate Blood Bank, Memphis, TN) in complete medium containing 10% human AB serum (heat inactivated) and 2.5% sodium bicarbonate (using 7.5% stock solution) in RPMI 1640 medium (KD Medical) supplemented with 50 μg/mL hypoxanthine and 25 mM HEPES. The culture flasks were grown in an incubator at 37°C and manually gassed with mixed gas (90% N_2_, 5% CO_2_, and 5% O_2_).

### Generation of the *piggyBac* mutant pilot-library.

The extensively characterized pilot library was used previously for multiple phenotypic screens in P. falciparum. It contains 128 isogenic *pB* mutants that were created during the course of our whole-genome random-mutagenesis saturation project (data are available in PlasmoDB [RRID:SCR_013331]). Briefly, the *piggyBac* mutant parasite clones were thawed individually and grown to 1 to 2% parasitemia in T25 flasks. Aliquots of the pilot library were generated by combining equal volumes of all clones and cryopreserving about 100 vials according to standard methods, ensuring a supply of enough biological replicate samples for use in phenotypic screens.

### Gametocyte pooled screen. (i) Assay design.

Figure 1 presents an overview of the gametocyte assay protocol that was employed in this study. Three biological replicates of the pilot library were thawed in nonvented T25 flasks (5 mL, 5% hematocrit, manual gassing, 37°C), and after reaching 1 to 2% parasitemia, the parasites were scaled up to 20-mL cultures in T75 flasks. With daily medium changes, the parasites were allowed to reach a higher parasitemia of 5 to 7%. Gametocyte induction was carried out by replacing half the medium at ~8% rings, and 24 h later, the trophozoite stages were split into multiple flasks at 0.5 to 0.8% parasitemia (15 mL, 5% hematocrit, manual gassing, 37°C [11, 82, 83]). Genomic DNA harvested from the induced flasks was sequenced by QI-seq on day 0. Following daily medium changes, a drop in hematocrit was introduced by increasing the volume of medium (~25 mL) at day 3. Gametocyte cultures were treated with 50 mM *N*-acetylglucosamine (NAG) from days 4 to 9 to eliminate any asexual parasites in the cultures and subjected to routine monitoring by using Giemsa-stained smears (84). When majority of the parasite population was stage V gametocytes on day 14, the mature stages were isolated by a Percoll gradient centrifugation and washed three times with incomplete medium (85). Subsequently genomic DNA was harvested using the Qiagen DNA extraction kit (QIAamp; catalog no. 51104).

### (ii) Phenotype identification.

Previously described QI-seq methodology was used to quantify insertion sites for each *piggyBac* mutant in the pool screened sample (40). Original read counts per insertion site were normalized using DEseq2 from R, and the fold change (day 14/day 0) for each mutant was calculated. The mutants were ranked from lowest to highest to identify phenotypes of interest. Hits at the bottom of the curve (log_2_ FC < −0.1) and at the top (log_2_ FC > 0.1) were categorized as hypoproducers and hyperproducers, respectively.

### (iii) Gene ontology enrichment analysis.

GO enrichment was performed by testing GO terms mapped to the gametocyte phenotypic categories of interest (hypoproducers, hyperproducers, and neutral) against a background of GO terms mapped to all other genes in the analysis using our R package pfGO (v 1.1) (86). The GO term database was created from the latest curated P. falciparum ontology available at the time of analysis from PlasmoDB, and enrichment was assessed via a weighted Fisher/elim-hybrid *P* value of ≤0.01 (v. 57). The fraction of genes represents the number of significant genes annotated to a given GO term in each category divided by the total number of genes annotated to a given GO term included in the analysis for all categories (background set).

### (iv) Evolutionary conservation pattern analysis.

To analyze the evolutionary conservation pattern of genes in the two phenotypic categories of interest, we used OrthoFinder to determine reciprocal best hits. The global alignment score (bit score) and median of the bit score (in cases of multicopy groups) were used to determine the percent identity in different *Plasmodium* species.

### Gametocyte cloned screen. (i) *pB* mutant clones: characteristics and validation.

The *piggyBac* transposon insertion sites of each *piggyBac* mutant clone were verified as previously described (30). The growth rates of individual *piggyBac* mutant clones were highly reproducible between biological replicates, as well as within the pilot library pool (41). Additionally, whole-genome sequencing performed on 29 of the 128 *piggyBac* mutant clones previously confirmed that no major genomic changes occurred aside from the *piggyBac* insertion in question, ensuring that the detected phenotypes were attributable to the single disruptions (38).

### (ii) Gametocyte conversion assay.

The sexual conversion assay was adapted from a well-established protocol (87) by using an abundant early sexual-stage marker, Pfg27 (87, 88). Parasite cultures were tightly synchronized by sorbitol synchronization, magnetically activated cell sorting (MACS) purification, and a second round of sorbitol synchronization. Subsequently, the 20- to 24-h-old parasites were diluted to 1% parasitemia at 2% hematocrit using culture medium consisting of 50% fresh and 50% conditioned medium – produced by incubation on the same strain and were then seeded in 96-well plates. After reinvasion, at 24 hpi, the medium was exchanged, and a 30-μL sample from each well was stained with SYBR green diluted 1:2,000 in RPMI and incubated at room temperature for 20 min, followed by two washes. Percent parasitemia was quantified by counting 50,000 events in the appropriate channels on an Accuri C6 flow cytometer. For gametocyte immunofluorescence staining, at approximately 64 hpi, a 150-μL sample from each well was fixed using a 4% paraformaldehyde–0.0075% glutaraldehyde solution in phosphate-buffered saline (PBS), permeabilized using 0.1% Triton X-100 in PBS, and then blocked in 3% bovine serum albumin (BSA) in PBS. Gametocytes were labeled using antibody to Pfg27 (kindly provided by Kim Williamson) and secondary Alexa Fluor 488 antibody, essentially as described previously (89). Percent gametocytemia was quantified either by counting 200,000 events on the fluorescein isothiocyanate (FITC) channel of an Accuri C6 flow cytometer or, for some clones, by counting stage II gametocytes per 20,000 red blood cells (RBC) on Diff-Quik-stained slides. For both parasitemia and gametocytemia readouts, control uninfected RBC wells incubated in the plate were used to subtract flow cytometry background. This experiment was performed with five replicates to determine average conversion rates [(% gametocytemia/% parasitemia) × 100] of that line. Clones with parasitemia that differed by more than ±1.5 standard deviations (SD) from the parasitemia of wild-type NF54 were not included in the phenotype analysis.

### Phenotypic characterization of hits. (i) Bioinformatic analysis.

Protein sequences of the mutant hits were obtained from PlasmoDB (accessed March 2022) and subjected to conserved domain prediction. The ExPASy Prosite database (https://prosite.expasy.org/) was used to identify putative localization signals, transmembrane regions and other bioinformatic characteristics.

### (ii) Morphological characterization.

The growth experiments and gametocytogenesis were performed with three biological replicates.

To compare asexual blood-stage parasite growth for the *piggyBac* mutant lines EMC3 and LRR5 and the parental wild-type NF54, sorbitol-synchronized ring-stage parasites were set at 0.25% initial parasitemia and followed for 2 growth cycles by using Giemsa-stained blood smears. The parasitemia was determined by counting at least 2,000 RBC under ×100 magnification. The gametocyte cultures were induced as previously described in our methods. Sixty microliters of culture was used to make smears for observation under light microscopy and determine gametocytemia (per 2,000 RBC) every other day. Stage distribution was determined by counting at least 50 gametocytes for each sample. An additional 60 μL was extracted from cultures, washed with incomplete medium, and used to prepare thin smears for immunofluorescence studies.

### (iii) IFAs.

Immunofluorescence assays (IFAs) were performed on air-dried smears fixed with 4% paraformaldehyde (PFA) for 10 min, followed by permeabilization with 0.1% Triton X-100 in PBS for 5 min, and blocked with 3% BSA in PBS blocking buffer overnight at 4°C. Incubation with primary antibodies was carried out for 1 h followed by secondary-antibody incubation for 45 min. The primary antibody used was PfGAP45 (rabbit, 1:5,000). The secondary antibody used was Alexa Fluor 488-conjugated goat anti-rabbit immunoglobulin (1:1,000) from Invitrogen. After incubation with primary and secondary antibodies, nuclei were stained with Hoechst dye for 10 min, and the slides were mounted with VectaShield Vibrance medium. The primary antibodies α-tubulin (mouse) and GAP45 (rabbit) were supplied by J.D. The secondary antibodies used were Alexa Fluor 594-conjugated goat anti-mouse immunoglobulin (1:1,000) and Alexa Fluor 488-conjugated goat anti-rabbit immunoglobulin (1:1,000) from Invitrogen. After incubation with primary and secondary antibodies, nuclei were stained with Hoechst dye for 10 min, and the slides were mounted with anti-fade Vectashield medium. The preparations were visualized under a Zeiss LSM 880 microscope with Airyscan 2 for high-resolution confocal images.

### Statistical analysis.

All statistical analyses were performed using the GraphPad Prism 9.00 statistical package. Pearson’s correlation coefficient was used to analyze consistency between biological replicates of the pooled screen. Other comparisons between wild-type and mutant parasite clones were analyzed using analysis of variance (ANOVA) and paired/unpaired *t* tests with appropriate corrections wherever necessary. Fisher’s exact test was used for protein conservation analysis.

### Data availability.

The mutants used in this study are available through the Malaria Research Reagent and Reference Repository (BEI Resources). Raw QI-seq data sets generated for this study were deposited in the European Nucleotide Archive under sample accession codes ERS3340839, ERS3340840, ERS8537954, ERS8537955, ERS8537958, ERS8537959, ERS8537956, ERS8537957, ERS8537960, and ERS8537961. Sample accession codes with their descriptions and processed QI-seq data are provided in Data Set S1.
