# Supplementary material for: Phenotypic Screens Identify Genetic Factors Associated with Gametocyte Development in the Human Malaria Parasite Plasmodium falciparum
Source: Microbiol Spectr. 2023 May 8;11(3):e04164-22. doi: 10.1128/spectrum.04164-22 (PMC10269797; doi:10.1128/spectrum.04164-22)
Supplement: Supplemental file 7 — Fig. S1 to S5. Download spectrum.04164-22-s0001.pdf, PDF file, 1.4 MB [file spectrum.04164-22-s0001.pdf]

Supplementary Figures

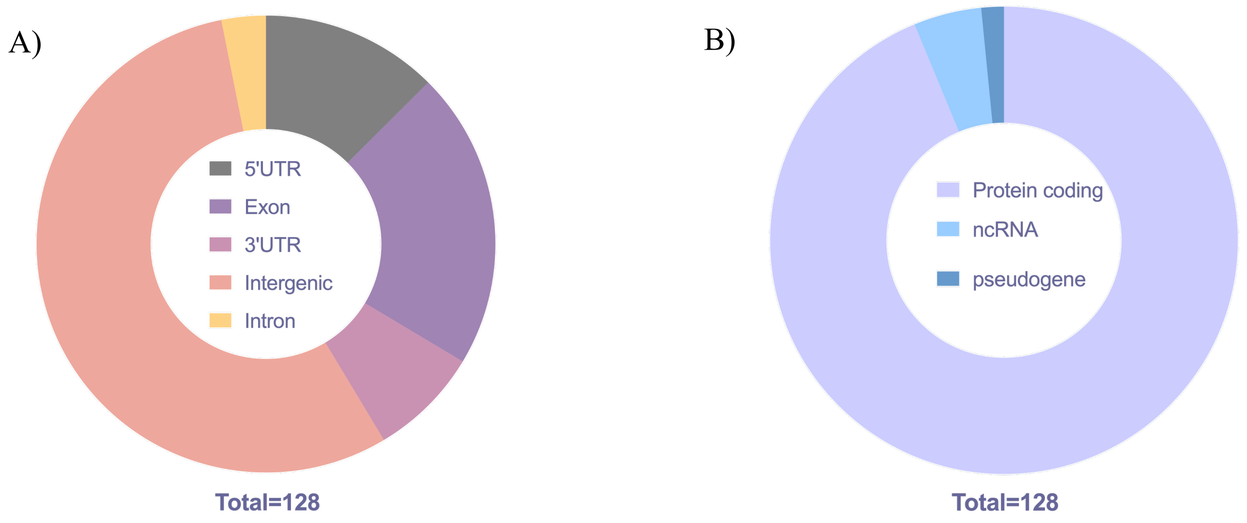

Fig. S1 Characteristics of the mutant-library used in the pooled screen (Dataset 1) **A)** Distribution of *piggyBac* insertions **B)** Distribution of type of genes in the pilot library

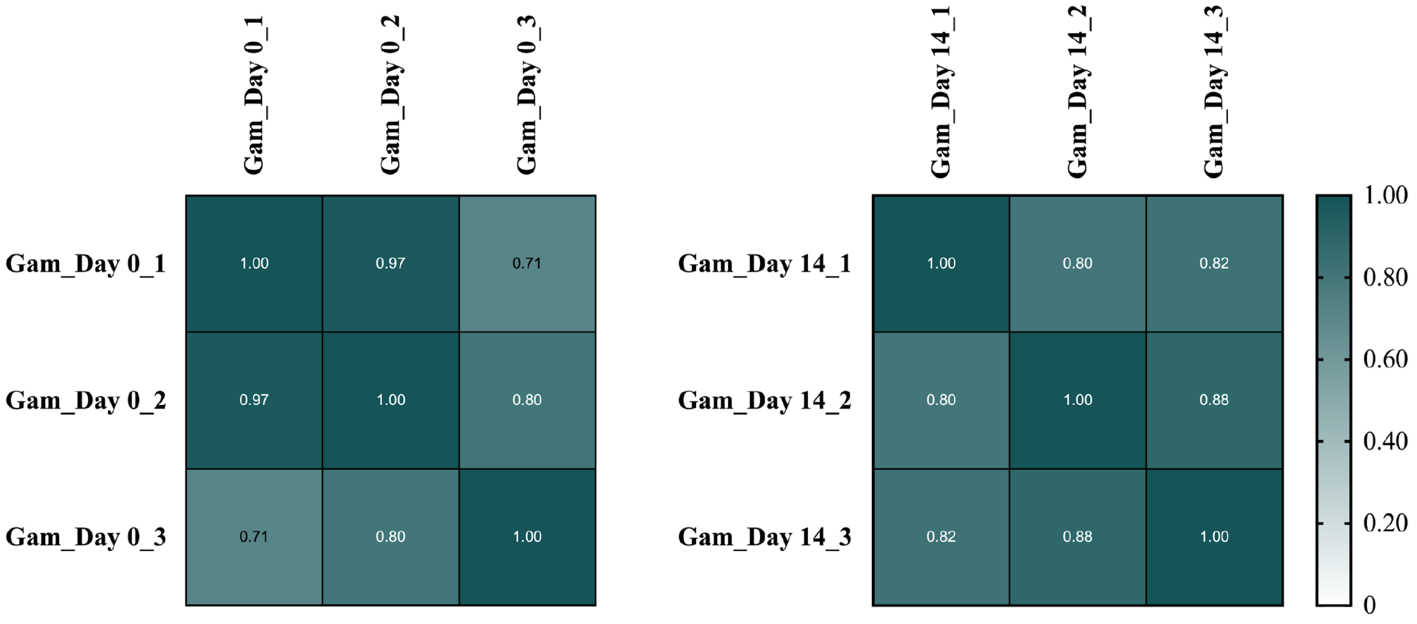

Fig. S2
High correlation between three biological replicates for normalized QIseq reads were observed for each time point demonstrating accuracy of sequencing data (Dataset 1).

A)

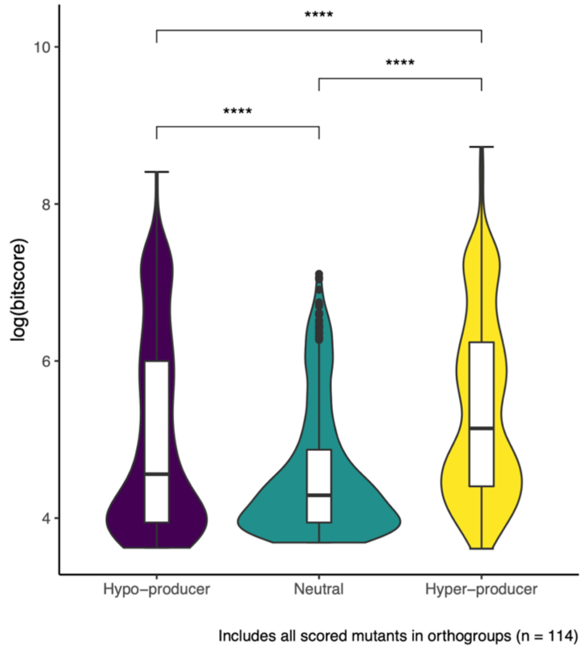

B)

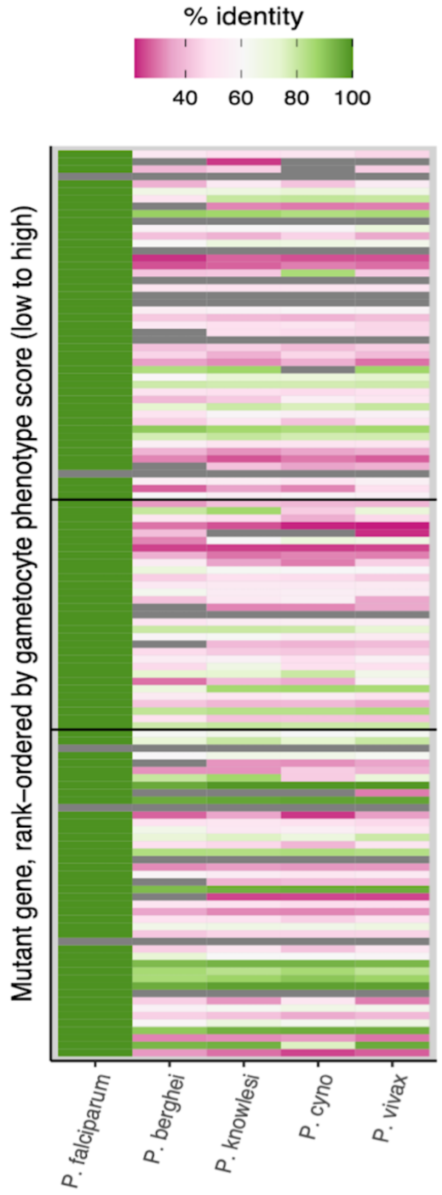

Fig. S3

A) Conservation across *Plasmodia* by gametocyte phenotype- Genes associated with a gametocyte phenotype are significantly conserved than those with a neutral phenotype. **B)** To analyze the evolutionary conservation pattern of genes in the two phenotypic categories of interest we used a reciprocal blast hit to obtain bit score and capture the percent identity in different *Plasmodium* species. High conservation was observed for majority of the genes in the phenotypic categories, except for 9 genes that had no orthologs and were specific to *P. falciparum* (Dataset 3) [1].

A)

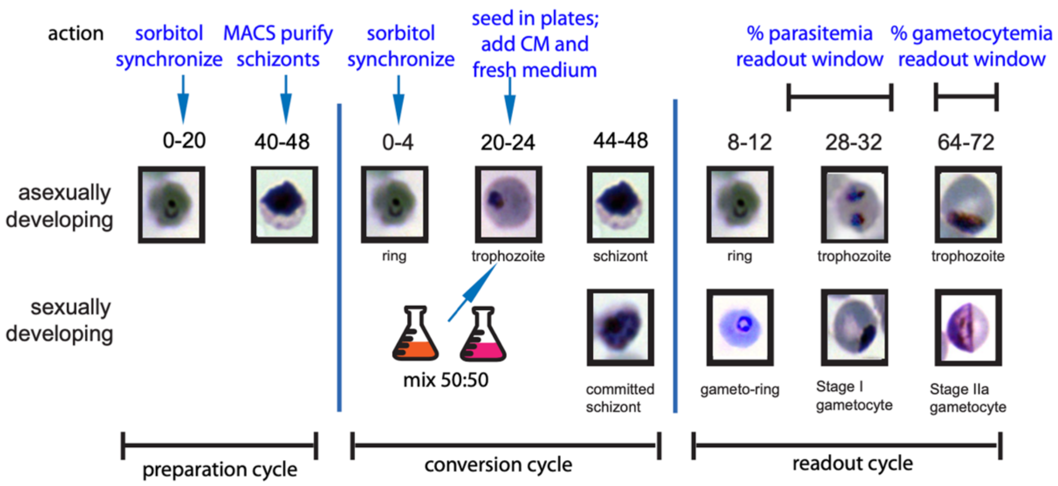

B)

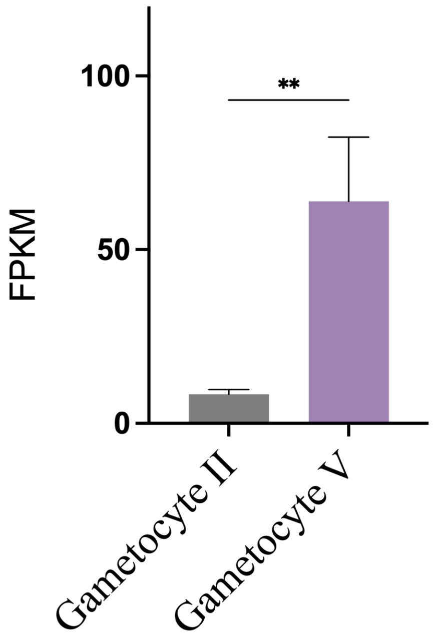

C)

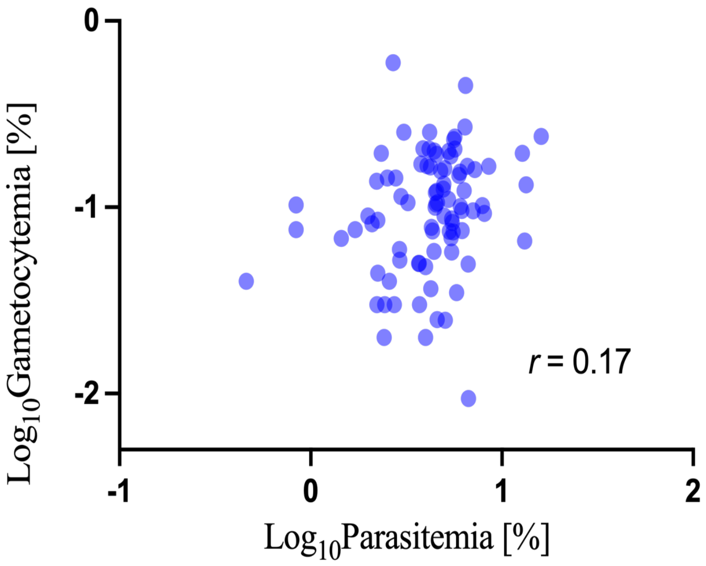

Fig. S4 A) Schematic timeline of the gametocyte conversion assay using individual mutant *piggyBac* clones. **B)** Genes corresponding to *piggyBac* mutants that had GCR > GCR of WT-NF54 had an increased expression in stage V gametocyte compared to stage II, implying they may be essential for later stages of gametocyte development. RNA seq data was obtained from Lopez Barragan *et al.* dataset (Dataset 2) [2].

C) Correlation between parasitemia and gametocytemia for all *piggyBac* mutants and controls obtained from the sexual conversion assay. Asexual parasitemia was weakly correlated with gametocytemia (Pearson r = 0.17) (Dataset 4).

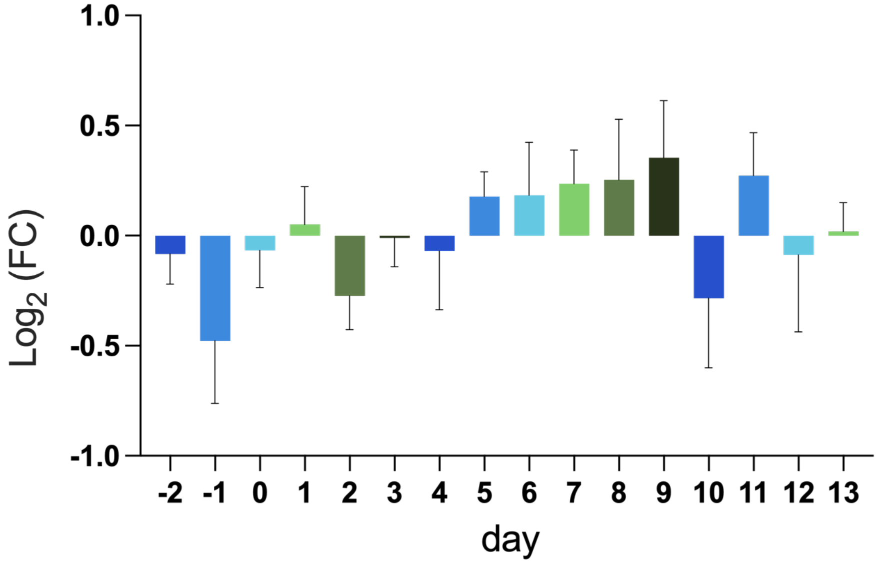

Fig. S5 Fine grain expression analysis of the top 15 gametocyte candidates observed in our study (Table 1). The expression values were obtained from Van Bilijon et. al (2019), where mRNA abundance was measured using microarray. (Dataset 6) [3]. Majority of the gametocyte genes are expressed from day 5 onwards of gametocyte development, with a small portion being expressed on day 1. (Data shown are mean and SD)

Supplementary References

1. Emms, D.M., Kelly, S. OrthoFinder: phylogenetic orthology inference for comparative genomics. Genome Biol 20, 238 (2019). https://doi.org/10.1186/s13059-019-1832-y
2. López-Barragán, M.J., et al., *Directional gene expression and antisense transcripts in sexual and asexual stages of Plasmodium falciparum.* BMC Genomics, 2011. **12**(1): p. 587.
3. Van Biljon, R., et al., *Hierarchical transcriptional control regulates Plasmodium falciparum sexual differentiation.* BMC Genomics, 2019. **20**(1).
